# Patient-Reported Mobility, Physical Activity, and Bicycle Use after Vulvar Carcinoma Surgery

**DOI:** 10.3390/cancers15082324

**Published:** 2023-04-16

**Authors:** Nick J. van de Berg, Franciscus P. van Beurden, G. C. Wanda Wendel-Vos, Marjolein Duijvestijn, Heleen J. van Beekhuizen, Marianne Maliepaard, Helena C. van Doorn

**Affiliations:** 1 Department of Gynaecological Oncology, Erasmus MC Cancer Institute, 3015 GD Rotterdam, The Netherlands; 2 Department of Biomechanical Engineering, Delft University of Technology, 2628 CD Delft, The Netherlands; 3 Centre for Nutrition, Prevention and Health Services, National Institute for Public Health and the Environment (RIVM), 3721 MA Bilthoven, The Netherlands

**Keywords:** vulvar carcinoma, patient-reported outcome, quality of life, mobility, physical activity, bicycling, EQ-5D-5L, SQUASH

## Abstract

**Simple Summary:**

For patients who are treated for vulvar cancer, walking and bicycling can become uncomfortable or painful. This can result in losses in mobility, physical activity, social contacts, self-reliance, and quality of life (QoL). We assessed the prevalence and severity of these problems using three questionnaires. In total, 84 patients who were treated for vulvar cancer between 2018 and 2021 participated. The reported QoL and perceived health were 0.832 ± 0.224 and 75.6 ± 20.0, respectively. Many patients indicated that saddle use has become painful, and that they experienced moderate or severe bicycling problems or could not bicycle (40.3%). Overall, patients treated for vulvar cancer reported a lower self-reported health, mobility, and physical activity, compared to baseline values found in the literature. The weekly time spent on walking, bicycling, and participating in sports was also reduced. This motivates us to investigate ways to reduce discomfort during physical activities, and help women regain their mobility and self-reliance.

**Abstract:**

Patients treated for vulvar carcinoma may experience losses in mobility and physical activity. In this study, we assess the prevalence and severity of mobility problems using patient-reported outcomes of three questionnaires: EQ-5D-5L to estimate QoL and perceived health; SQUASH to estimate habitual physical activity; and a problem-specific questionnaire on bicycling. Patients treated for vulvar carcinoma between 2018 and 2021 were recruited, and 84 (62.7%) responded. The mean age was 68 ± 12 years (mean ± standard deviation). Self-reported QoL and perceived health were 0.832 ± 0.224 and 75.6 ± 20.0, respectively. Dutch physical activity guidelines were met by 34.2% of participants. Compared to baseline values, the times spent walking, bicycling, and participating in sports were all reduced. During bicycling, patients experienced moderate or severe pain in the skin of the vulva (24.5%), pain in the sit bones (23.2%), chafing (25.5%), or itching (8.9%). Overall, 40.3% experienced moderate or severe bicycling problems or could not bicycle, 34.9% felt that their vulva impeded bicycling, and 57.1% wished to make more or longer bicycling journeys. To conclude, vulvar carcinoma and its treatment reduce self-reported health, mobility, and physical activity. This motivates us to investigate ways to reduce discomfort during physical activities, and help women regain their mobility and self-reliance.

## 1. Introduction

Bicycling is an integral part of Dutch life and a popular mode of transportation around the world. In the Netherlands, it accounts for nearly one quarter of all journeys [1]. As an urban traffic alternative, it is affordable, flexible, social, and sustainable, and can potentially reduce traffic congestions and air and noise pollution. If everyone were to bicycle as much as the Dutch, this would be equivalent to a 20% reduction in carbon emissions based on the global passenger car fleet of 2015 [2]. In short, enabling people to bicycle is of societal value. This is also recognized and becomes visible by the multitude of bicycle-sharing systems and bicycle highways introduced in major cities around the world [3,4]. Furthermore, the advent of e-bikes has increased the accessibility of this mode of transportation for the elderly and less mobile. 

Physical activity and bicycling are associated with substantial health benefits [5]. In the Netherlands in 2020, the average bicycling time for elderly in the age groups 65+ and 85+ was 210 and 38 min/week, respectively [6]. This means bicycling contributes significantly to the ability to reach physical activity guidelines, which includes a recommended engagement in (moderately intense) physical activity for 150 min/week. At the same time, the inability to bicycle may impact quality of life (QoL) as bicycling can improve mobility and self-reliance, e.g., the ability to go out for groceries or to reach friends or family. Finally, the impact can be emotional, in terms of the ability to feel young.

In oncology, increased physical activity can help prevent cancer and improve health and QoL outcomes [7,8,9]. Conversely, cancer and its treatments can impact the ability to be physically active. For patients with vulvar (pre)malignancies, both walking and bicycling can become painful. Some patients decide to quit bicycling altogether. Currently, data on the prevalence of these impediments are not available. Neither do we know whether existing solutions to increase bicycling comfort, e.g., saddles with cut-outs [10], are effective for this target group.

In this study, we evaluate how vulvar carcinoma and its surgical treatment impacts mobility, physical activity, and the ability to bicycle. In our study, mobility and physical activity are addressed using the validated EQ-5D-5L and SQUASH questionnaires, while the ability to bicycle is addressed using a non-validated problem-specific Gynaecological Oncology-Bicycling (GO-Bicycling) questionnaire.

## 2. Materials and Methods

### 2.1. Study Population

Participants were recruited by approaching patients who were treated for vulvar carcinoma at the Erasmus MC Cancer Institute between 2018 and 2021. Recruitment took place between March and May 2022. To study outcomes of patients in the day-to-day mix of vulvar cancer types and stages treated in our hospital, no clinical exclusion criteria were formulated. All surviving patients were included, except for patients who emigrated (n = 2), were unable to read and write Dutch (n = 1), or had dementia (n = 1). The ability or desire to bicycle played no part in the recruitment. Patients were first approached via e-mail, with a link to the digital questionnaires created in our data management platform (Castor EDC, Amsterdam, the Netherlands). A reminder was sent two and a half weeks after this e-mail. Finally, a printed version of the questionnaires was sent by conventional mail to patients who had not yet responded. This was needed, as the mean age of the target group was relatively high, and some patients were digitally inactive. In total, 134 patients were approached. This study was approved by the Medical Ethics Review Committee of the Erasmus MC (protocol: MEC-2022-0077, date of approval: 17-02-2022). All participants included in this study received a patient information letter, providing background information on study goals, the expected questionnaire completion time, that participation is voluntary, and about data management and protection of privacy. All participants gave consent before filling in the questionnaires.

### 2.2. Patient-Reported Outcome Instruments

Participants completed three questionnaires, of which two were validated. Their data were processed according to conventions [11,12]. Data of the non-validated questionnaire were not processed. The results are shown directly using summary statistics. 

#### 2.2.1. EQ-5D-5L Questionnaire

The EQ-5D-5L questionnaire assesses QoL as a combination of patient-reported scores for mobility, self-care, usual activities, pain/discomfort, and anxiety/depression. Each factor has five levels: no problems, slight problems, moderate problems, severe problems, and extreme problems. This creates 3125 (=55) health states, which can be translated to a single *QoL index*: *I_QoL_* ≤ 1. This was achieved using the constraint tobit model with Dutch tariffs [11], according to the penalty function Equation (1):

(1) $$I_{QoL}=1-c_{0}-\beta_{i}^{MO}-\beta_{i}^{SC}-\beta_{i}^{UA}-\beta_{i}^{PD}-\beta_{i}^{AD},$$

Here, *c*_0_ = 0.047 is a constant penalty for all health states except 11111, and $\beta_{i}^{MO},\;\beta_{i}^{SC},\;\beta_{i}^{UA},\;\beta_{i}^{PD},\;\beta_{i}^{AD}$ are the level (*i*) dependent penalties for the five evaluation domains. Thus, *I_QoL_* = 1 is the highest QoL score possible. In addition, the EQ-VAS extension provided an overall patient-reported *health index*, based on a 100-point visual analogue scale (VAS). Here, 0 is the worst imaginable health, and 100 the best imaginable health.

#### 2.2.2. SQUASH Questionnaire

The Short Questionnaire to Assess Health-enhancing physical activity (SQUASH) provides a patient-reported measure of the amount of habitual physical activity in an average week. It addresses the duration and intensity of activities in the domains: commuting, leisure, household, and work or school [12]. Using this questionnaire, the percentage of the population that adheres to Dutch physical activity guidelines can be calculated [13]. To comply with the guidelines, two criteria must be met. First, a person needs to engage in a minimum of 150 min of at least moderately intense physical activity per week. Secondly, a person needs to perform at least two activities per week that are bone and muscle strengthening.

For the first criterion, an estimate of total physical activity (min/week) was calculated by addition of the times spent in each SQUASH category. Here, household activities and activities at work were excluded, as they were considered low intensity activities [14]. Activities with a metabolic equivalent of task (MET) score of at least 3.0 were considered to be of at least moderate intensity.

#### 2.2.3. GO-Bicycling Questionnaire

The problem-specific questionnaire collects data on relations between vulvar carcinoma and its treatment on bicycle use. First, the types of bicycles and support tools used are registered. Subsequently, the questionnaire assesses the ability and desire to make use of bicycles. In addition, the types and intensities of pain/discomfort that are experienced during bicycling are registered. Scores are collected on pain in the skin of the vulva, pain in the sit bones, chafing, and itching. Scores are expressed with a Likert scale, using the levels: no, slight, moderate, and severe, both during and after bicycle use.

### 2.3. Data Analysis

The dataset and questionnaire (Dutch) can be found online as Supplementary Material (supplementary files). Statistical analyses were performed in MATLAB R2021A (Mathworks, Natick, MA, USA) and SPSS 28.0.1.1 (IBM, Armonk, NY, USA). The *QoL index* (EQ-5D-5L) and the *health index* (EQ-VAS) were compared to population norms using a one-sample Wilcoxon signed-rank test with significance level α = 0.05. Comparisons are illustrated using bar graphs with error bars, indicating the standard deviation (S.D.). Graphs indicate the number of respondents per question (n). Furthermore, we determined the strength of linear correlations between *age*, the *health index*, and the *QoL index*, using a Pearson correlation coefficient. The linear fit was derived with a least squares first order polynomial function. The remainder of the comparisons are descriptive. They are illustrated with stacked bar charts, indicating the percentage of respondents with answers in each level.

## 3. Results

### 3.1. Study Population

Of the 134 patients approached, 84 returned their questionnaires (response rate: 62.7%). Most respondents completed the three questionnaires (60/84). Of partially completed questionnaires, on average 62.1% of the questions were answered. Table 1 provides a brief overview of patient characteristics, including age and the history of surgery and radiotherapy. The age of respondents was 68 ± 12 (mean ± S.D.) years, compared to 68 ± 17 years for non-respondents. Of the people that were able to bicycle, 50.0% owned a city bike and 46.4% an electric bike. In total, 31.7% has made at least one type of bicycle adjustment to reduce pain or discomfort.

### 3.2. Patient-Reported Outcomes

**Figure 1:**
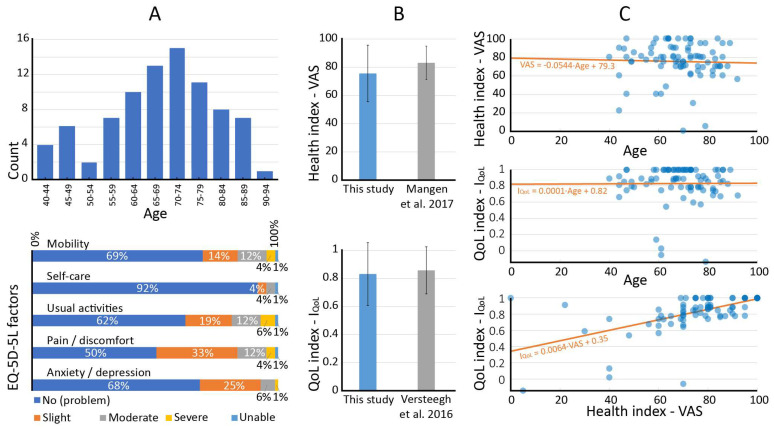
Population age histogram (n = 84) and EQ-5D-5L QoL factors presented as stacked bar graphs (**A**). Reported overall *QoL* and *health* are shown next to Dutch baseline values (**B**) [11,15]. Relations between *age*, the *QoL index* and the *health index* are shown with scatter plots and a linear fit line (**C**).

An overview of patient-reported outcomes and corresponding Dutch baseline values is provided in Table 2. Results of the EQ-5D-5L and EQ-VAS are shown in Figure 1. It was found that the patient-reported *health index* (75.6 ± 20.0) was lower than the baseline value (*p* = 0.0025). No difference was found in the overall *QoL index* (0.832 ± 0.224), compared to the baseline value (*p* = 0.44). Patient-reported mobility problems (any) were reported by 31.0% (26/84) of respondents. The linear least squares fits and Pearson correlation coefficients showed a correlation between the *QoL index* and the *health index* (*p* = 0.573). No clear correlations were seen between the *health index* and *age* (*p* = −0.033), or between the *QoL index* and *age* (*p* = 0.006).

The SQUASH questionnaire showed that physical activity guidelines were met by 34.2% (27/79). The first criterion for adhering to the physical activity guidelines (150 min/week of moderate intensive activities) was met by 36.7% (29/79) of respondents. The second criterion for adhering to the physical activity guidelines (bone and muscle strengthening activities) was met by 82.3% (65/79) of respondents. The share of respondents that used a bicycle at least one time per week was 48.1% (38/79), and the share that participated in sports at least one time per week was 30.4% (24/79). Respondents spent 75 ± 192 min/week gardening and 28 ± 97 min/week doing odd jobs. Compared to baseline values (Table 2), vulvar carcinoma patients spent less time walking (240 ± 303 min/week, *p* < 0.0001), bicycling (96 ± 169 min/week, *p* < 0.0001), and participating in sport activities (48 ± 129 min/week, *p* < 0.0001).

**Figure 2:**
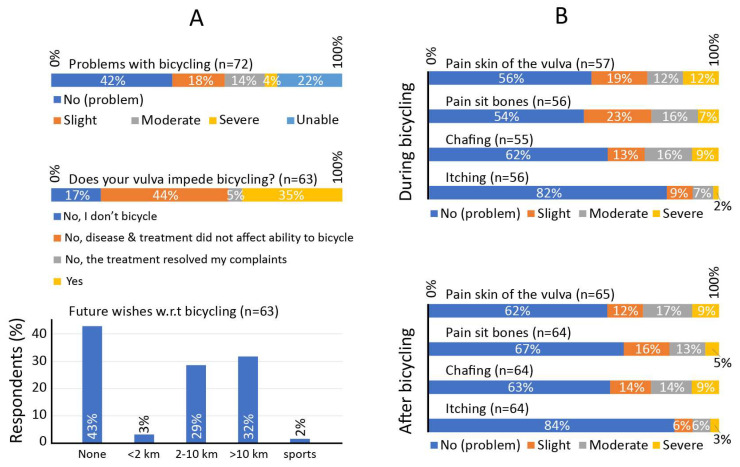
Results of the GO-Bicycling questionnaire, showing the prevalence of problems with bicycling, their relation to problems of the vulva (**A**-top), and the desire to bicycle more (**A**-bottom). In addition, patient-reported pain or discomfort is reported during and after bicycle use (**B**).

Finally, the GO-Bicycling questionnaire results are summarized in Figure 2. In total, 40.3% of respondents experienced moderate to severe problems with bicycling (18.1%, 13/72) or were unable to bicycle (22.2%, 16/72). When asked whether the bicycling impediment could be related to the vulva, 34.9% (22/63) indicated this was the case. Respondents with no or slight bicycling problems could bicycle a distance of 19 ± 17 km, and time of 73 ± 53 min. In turn, respondents with moderate to severe problems could bicycle 9 ± 16 km (Wilcoxon rank sum test: *p* = 0.007), and time of 37 ± 68 min (Wilcoxon rank sum test: *p* = 0.003). Overall, 57.1% (36/63) of respondents had a desire to make more or longer bicycling journeys, and 31.7% (20/63) has tried at least one aid or adaptation to improve comfort, e.g., special saddles or bicycle settings (see Table 1). Of the remaining group, 57.1% (24/42) indicated that they were unaware of the existence of such aids.

Types of pain and discomfort experienced by vulvar carcinoma patients are presented in Figure 2B. Often-reported complaints during bicycling included moderate to severe pain in the skin of the vulva (24.5%, 14/57), pain in the sit bones (23.2%, 13/56), and chafing (25.5%, 14/55), whereas itching was reported less frequently (8.9%, 5/56). The prevalence of pain in the sit bones was higher during bicycling than after bicycling.

## 4. Discussion

In this study, we assessed mobility, physical activity, and bicycle use in women treated for vulvar carcinoma. This was achieved with patient-reported outcomes of three questionnaires: EQ-5D-5L with EQ-VAS, SQUASH, and a problem-specific GO-Bicycling questionnaire. When comparing our EQ-5D results to baseline values reported in the literature, patient-reported mobility problems (any) were experienced twice as often (31.0% vs. 16.0%), the VAS *health index* was reduced (75.6 vs. 83.2), and the *QoL index* was similar (0.832 vs. 0.858).

The SQUASH results provided insight into the way patient mobility was affected. Physical activity guidelines were met by 34.2% of participants, which is considerably lower than the baseline value of 46.5% (see Table 2). Compared to 65–69 Dutch women, the number of minutes per week walking (240 vs. 367 min), bicycling (96 vs. 194) and sporting (48 vs. 123) were also reduced. 

The GO-Bicycling questionnaire showed that approximately 1/3rd of respondents felt that their vulva impeded bicycling. The most often reported problems were pain in the skin of the vulva, pain in the sit bones, and chafing. Respondents with ‘no or slight’ and ‘moderate or severe’ bicycling problems indicated that they could bicycle continuously for 73 min and 37 min, respectively. 

Although 40.3% of respondents experienced moderate or worse problems with bicycling, 31.0% experienced moderate or worse problems with mobility. This suggests that bicycling problems do not translate to mobility problems for all patients, i.e., bicycling may be replaced by walking, driving, or using public transport. Nevertheless, more than half of the respondents wished to bicycle more. The effectiveness of bicycle adaptations or aids to improve comfort may be patient-specific. However, most are harmless to try. They may include use of (chamois) cream, optimized bicycle fit and posture, special bib shorts [16], or saddles with cut-outs [10].

A limitation of this study is the lack of validation and baseline values for the problem-specific questionnaire. In terms of question formulations, we aimed to adhere to word choices used in the EQ-5D-5L and SQUASH questionnaires, e.g., “think about an average week in the past months”, and “I have moderate problems with bicycling” as one of the answer levels. Even in the literature, reference values for problems with functional bicycling are currently still missing. Numerous studies address injuries caused by sports or bicycle overuse, but these likely differ in location and prevalence, and are generally assessed in a much younger population. For the validated questionnaires, baseline values were used from subpopulations with the closest resemblance to our own population, i.e., healthy women with a same mean age. Nevertheless, groups did differ, e.g., the range of ages in our population (40–92 years) was typically larger compared to that in baselines. This could have impacted the analysis. 

Clinically, we decided to look at the whole population. It would be interesting to research bicycling problems in vulvar carcinoma patient subgroups, e.g., by stratifying for tumor location and size, primary or recurrent disease, treatment of groin lymph nodes, reconstructive surgery, exclusive or adjuvant radiotherapy, or the incidence of lymphedema. As many of these factors may interact, the creation of balanced and sufficiently large subgroups would require a multi-center prospective study. In such a study, questionnaires may be completed before and after surgery to delineate disease-related and treatment-related problems. Bicycling discomfort may differ per subgroup, and understanding these relations may result in finding effective and personalized solutions. 

Our study shows that mobility and bicycling problems can arise even with tumors that are ≤ 2cm in size (70%) or treatments that consist solely of surgery (83%). It should be realized that patients with recurrent disease or other ongoing vulvar skin conditions, such as lichen sclerosus, were not excluded in our study. Vulvar cancer is characterized by life-long follow-up, gradual disease development and high recurrence rates for at least 10 years [17]. The consideration of post-surgical problems in ‘healthy’ patients only would yield a test group that is not a cross-section of patients seen in our hospital. 

Our results may be affected by a non-response bias. However, we are uncertain of the directional tendency. Possibly, there is a slight overestimation of problems in our reported results. Non-responders may be uninvested in the topic, because they do not bicycle, do not experience problems, or because they feel the questions are too personal. However, we are pleased with the response rate (63%), and expect that the impact of such a bias would be small. In addition, a limited group of respondents (n = 11) inadvertently received a version of the questionnaire in which levels of pain and discomfort were inquired ‘before’ and ‘after’ bicycling, instead of ‘during’ and ‘after’. This was the only difference between questionnaires. We decided to include and show all data, with the exception of these 11 responses on this one question. This explains why more patients reported problems after bicycling, compared to during bicycling in Figure 2.

Finally, we need to remark that mobility, physical activity, and QoL responses may have been affected by the COVID-19 pandemic. From the Health Survey/Lifestyle Monitor, Statistics Netherlands (CBS) in collaboration with National Institute for Public Health and the Environment (RIVM), it appeared that physical activity guidelines were met by more people (65+) in 2020–2021, compared to in the pre-COVID-19 years [6]. This might however be partly due to methodological issues related to the pandemic, such as the impact of lock-downs on face-to-face interviews and the impact of the pandemic such as on interpretations of concepts of habitual physical activity levels. In the literature, it was shown that time spent walking and doing odd jobs increased during lockdowns, while time spent exercising declined [18]. Overall, it has also been shown that physical activity decreased during lockdown [19]. However, our understanding of lasting (post-lockdown) effects on physical activity remains limited.

## 5. Conclusions

Our study quantified the prevalence and severity of mobility and physical activity problems among vulvar carcinoma patients. Compared to Dutch baseline values, we found: (1) a reduced *health index* (75.6 ± 20.0 vs. 83.2 ± 11.8), (2) a similar overall QoL (0.832 ± 0.224 vs. 0.858 ± 0.168), (3) doubling of mobility problems (31.0% vs. 16.0%), (4) reduced physical activity (34.2% vs. 46.5%), and (5) a strong reduction in bicycling time (96 vs. 194 min/week). Moderate or worse bicycling problems are experienced frequently (40.3%). Both during and after bicycling, pain or discomfort can be experienced in the skin of the vulva or near the sit bones, or caused by local chafing. These results motivate our team to investigate ways to increase bicycling comfort and help our patients improve their physical activity, mobility, and self-reliance.

## Figures and Tables

**Table 1:** Overview of characteristics and medical history of vulvar carcinoma patients. In addition, the table shows bicycle ownership, and previous attempts to reduce bicycling discomfort or pain.

**Electronic Health Records**			
Tumor type		Groin involvement (if treatment was surgery)	
Squamous cell carcinoma	94%	None	16%
Melanoma	5%	Unilateral sentinel node	21%
Adenocarcinoma	1%	Unilateral lymphadenectomy	9%
		Bilateral sentinel node	40%
Tumor size		Bilateral lymphadenectomy	10%
≤2 cm	70%	Bilateral sentinel node + lymphadenectomy	4%
>2 cm	30%		
		
Tumor location		**Self-reported data**	
Unilateral > 1 cm	17%	Age (range: 40–92 years):	
Near vulva midline ≤ 1 cm	65%	<65	29 (34.5%)
Bilateral (midline involved)	18%	≥65	55 (65.5%)
			
Tumor stage		Bicycles owned (n = 56) ^2^:	
I	74%	City bike	28 (50.0%)
II	5%	Electrical bike	26 (46.4%)
III	20%	Mountain bike	2 (3.6%)
		Race bike	0 (0.0%)
Treatment		Other ^3^	4 (7.1%)
Surgery	83%		
Surgery + adjuvant radiotherapy ^1^	8%	Attempts to reduce bicycling problems (n = 63) ^2^:	
Primary chemo-radiotherapy	6%	None	43 (68.3%)
Primary radiotherapy	3%	New bike	3 (4.8%)
		New saddle	14 (22.2%)
Surgery type (if treatment was surgery)		Bike-fitting/bike adjustments	4 (6.3%)
Local	3%	Bicycle clothing	3 (4.8%)
Wide local / re-excision	96%	Other	2 (3.2%)
With plastic surgery	1%		

^1^ To the groins, vulva, or both. ^2^ Respondents could provide more than one answer: the numbers do not add up to 100%. ^3^ For example, recumbent bicycles or tricycles; two respondents indicated that they use a home trainer.

**Table 2:** Overview of Dutch baseline values and patient-reported outcomes (PRO) of the validated questionnaires in this study.

Metric	Baseline Population	Baseline Value	PRO
**EQ-5D-5L**			
QoL index	Dutch women, all ages [11]	0.858 ± 0.168	0.832 ± 0.224
Mobility problems (any)	Dutch women, 65–69 years [15]	16.0%	31.0%
Health index (VAS)	Dutch women, 65–69 years [15]	83.2 ± 11.8	75.6 ± 20.0
**SQUASH**			
Physical activity guideline, total	Dutch women, 65–69 years [6]	46.5%	34.2%
Physical activity criterion 1: min/week	Dutch women, 65–69 years [6]	52.4%	36.7%
Physical activity criterion 2: muscle/bone strengthening	Dutch women, 65–69 years [6]	84.5%	82.3%
Weekly bicycle use	Dutch women, 65–69 years [6]	64.4%	48.1%
Weekly sport participation	Dutch women, 65–69 years [6]	40.9%	30.4%
Walking total, min/week	Dutch women, 65–69 years [6]	367 ± 387	240 ± 303
Bicycling total, min/week	Dutch women, 65–69 years [6]	194 ± 257	96 ± 169
Sport participation total, min/week	Dutch women, 65–69 years [6]	123 ± 261	48 ± 129

## Data Availability

The dataset used in this study is added as an online additional file.

## Supplementary Materials

The following supporting information can be downloaded at: https://www.mdpi.com/article/10.3390/cancers15082324/s1, Supplementary files: GO-Bicycling questionnaire (Dutch), dataset.

## References

[1] Pucher J., Buehler R. (2008). Cycling for Everyone Lessons from Europe. Transport. Res. Rec..

[2] Chen W., Carstensen T.A., Wang R.R., Derrible S., Rueda D.R., Nieuwenhuijsen M.J., Liu G. (2022). Historical patterns and sustainability implications of worldwide bicycle ownership and use. Commun. Earth Environ..

[3] Ma X.W., Yuan Y.F., Van Oort N., Hoogendoorn S. (2020). Bike-sharing systems’ impact on modal shift: A case study in Delft, the Netherlands. J. Clean. Prod..

[4] Agarwal A., Ziemke D., Nagel K. (2020). Bicycle superhighway: An environmentally sustainable policy for urban transport. Transp. Res. Part A Policy Pract..

[5] Oja P., Titze S., Bauman A., de Geus B., Krenn P., Reger-Nash B., Kohlberger T. (2011). Health benefits of cycling: A systematic review. Scand. J. Med. Sci. Sports.

[6] CBS, RIVM (2021). National Health Survey/Lifestyle Monitor.

[7] McTiernan A., Friedenreich C.M., Katzmarzyk P.T., Powell K.E., Macko R., Buchner D., Pescatello L.S., Bloodgood B., Tennant B., Vaux-Bjerke A. (2019). Physical Activity in Cancer Prevention and Survival: A Systematic Review. Med. Sci. Sport. Exerc..

[8] Campbell K.L., Winters-Stone K.M., Wiskemann J., May A.M., Schwartz A.L., Courneya K.S., Zucker D.S., Matthews C.E., Ligibel J.A., Gerber L.H. (2019). Exercise Guidelines for Cancer Survivors: Consensus Statement from International Multidisciplinary Roundtable. Med. Sci. Sport. Exerc..

[9] Sprod L.K., Mohile S.G., Demark-Wahnefried W., Janelsins M.C., Peppone L.J., Morrow G.R., Lord R., Gross H., Mustian K.M. (2012). Exercise and Cancer Treatment Symptoms in 408 Newly Diagnosed Older Cancer Patients. J. Geriatr. Oncol..

[10] Larsen A.S., Larsen F.G., Sorensen F.F., Hedegaard M., Stottrup N., Hansen E.A., Madeleine P. (2018). The effect of saddle nose width and cutout on saddle pressure distribution and perceived discomfort in women during ergometer cycling. Appl. Ergon..

[11] Versteegh M.M., Vermeulen K.M., Evers S.M.A.A., de Wit G.A., Prenger R., Stolk E.A. (2016). Dutch Tariff for the Five-Level Version of EQ-5D. Value Health.

[12] Wendel-Vos G.C.W., Schuit A.J., Saris W.H.M., Kromhout D. (2003). Reproducibility and relative validity of the Short Questionnaire to Assess Health-enhancing physical activity. J. Clin. Epidemiol..

[13] Weggemans R.M., Backx F.J.G., Borghouts L., Chinapaw M., Hopman M.T.E., Koster A., Kremers S., van Loon L.J.C., May A., Mosterd A. (2018). The 2017 Dutch Physical Activity Guidelines. Int. J. Behav. Nutr. Phys. Act..

[14] Gal R., Monninkhof E.M., Peeters P.H.M., van Gils C.H., van den Bongard D.H.J.G., Wendel-Vos G.C.W., Zuithoff N.P.A., Verkooijen H.M., May A.M. (2019). Physical activity levels of women with breast cancer during and after treatment, a comparison with the Dutch female population. Acta Oncol..

[15] Mangen M.J., Bolkenbaas M., Huijts S.M., van Werkhoven C.H., Bonten M.J., de Wit G.A. (2017). Quality of life in community-dwelling Dutch elderly measured by EQ-5D-3L. Health Qual. Life Outcomes.

[16] Larsen A.S.T., Norheim K.L., Marandi R.Z., Hansen E.A., Madeleine P. (2021). A field study investigating sensory manifestations in recreational female cyclists using a novel female-specific cycling pad. Ergonomics.

[17] te Grootenhuis N.C., van der Zee A.G.J., van Doorn H.C., van der Velden J., Vergote I., Zanagnolo V., Baldwin P.J., Gaarenstroom K.N., van Dorst E.B., Trum J.W. (2016). Sentinel nodes in vulvar cancer: Long-term follow-up of the GROningen INternational Study on Sentinel nodes in Vulvar cancer (GROINSS-V) I. Gynecol. Oncol..

[18] van Bakel B.M.A., Bakker E.A., de Vries F., Thijssen D.H.J., Eijsvogels T.M.H. (2021). Impact of COVID-19 lockdown on physical activity and sedentary behaviour in Dutch cardiovascular disease patients. Neth Heart J..

[19] Schoofs M.C.A., Bakker E.A., de Vries F., Hartman Y.A.W., Spoelder M., Thijssen D.H.J., Eijsvogels T.M.H., Buffart L.M., Hopman M.T.E. (2022). Impact of Dutch COVID-19 restrictive policy measures on physical activity behavior and identification of correlates of physical activity changes: A cohort study. BMC Public Health.

