# Supplementary material for: Patient-Reported Mobility, Physical Activity, and Bicycle Use after Vulvar Carcinoma Surgery
Source: Cancers (Basel). 2023 Apr 16;15(8):2324. doi: 10.3390/cancers15082324 (PMC10136582; doi:10.3390/cancers15082324)
Supplement: Supplementary file 1 [file cancers-15-02324-s001.zip › Appendix A GO-bicycling questionnaire.pdf]

Appendix A: GO-Bicycling questionnaire (Dutch)

Neemt u nu weer een normale week in de afgelopen maanden in gedachten. In het volgende deel van de vragenlijst willen we met u nagaan in hoeverre de vulva/huidproblemen en de behandeling effect heeft gehad op fietsen.

Fietsen in het algemeen

Ik heb geen problemen met fietsen	☐
Ik heb een beetje met fietsen	☐
Ik heb matige problemen met fietsen	☐
Ik heb ernstige problemen met fietsen	☐
Ik ben niet in staat om te fietsen	☐

Kunt u aangeven in tijd en/of afstand hoever u kunt fietsen.
(getal) uren, (getal) minuten, (getal) afstand in kilometers.

Kunt u aangeven op wat voor een soort fiets u fietst.

Gewone stadsfiets	☐
Elektrische fiets	☐
Racefiets	☐

Wij weten dat veel vrouwen verschillende klachten ervaren tijdens of na het fietsen.

Kunt u hieronder aangeven hoe u de volgende klachten ervaart?

Tijdens het fietsen:

Pijn in de zitbotjes	(geen, een beetje, matig, ernstig)
Pijn in de huid van de schede/vulva	(geen, een beetje, matig, ernstig)
Jeuk	(geen, een beetje, matig, ernstig)
Schurend gevoel	(geen, een beetje, matig, ernstig)

Andere klachten, namelijk: …………………………

Na het fietsen:

Pijn in de zitbotjes	(geen, een beetje, matig, ernstig)
Pijn in de huid van de schede/vulva	(geen, een beetje, matig, ernstig)
Jeuk	(geen, een beetje, matig, ernstig)
Schurend gevoel	(geen, een beetje, matig, ernstig)

Andere klachten, namelijk: …………………………

Wordt u door de vulva belemmert in het fietsen?

Nee, ik fietste voor de behandeling ook niet. ☐
Nee, ik kan nog net zo goed fietsen als voor deze ziekte en behandeling. ☐
Nee, door de behandeling is de belemmering verminderd of verholpen ☐
Ja ☐

Indien ja:
Ik zou deze activiteiten in de toekomst vaker willen doen:
(meerdere antwoorden mogelijk)

Fietsen tot 2 km ☐
Korte fietstochten, 2 – 10 km ☐
Lange fietstochten, > 10 km ☐
Sporten op de fiets ☐

Ook zijn wij benieuwd of u bekend bent met – en gebruik maakt van – hulpmiddelen voor het fietsen, zoals een speciaal zadel, andere afstelling of andere fiets.
Heeft u aanpassingen gedaan in het fietsen?

Nee ☐
Ja, namelijk: ……………… ☐

Indien nee:
Waarom niet?

Ik wist niet van deze hulpmiddelen af ☐
Ik wist dat deze hulpmiddelen bestonden, maar niet hoe ik hier aan moest komen ☐
Ik wist dat deze hulpmiddelen bestonden, maar dit is me de moeite niet waard ☐
Anders, namelijk: ………….. ☐

Er zijn speciale fietszadels op de markt om de druk op de vulva te verminderen.
Hoeveel zou u bereid zijn hier aan uit te geven?

0 - 50€ ☐
50€ - 100€ ☐
100€ - 150€ ☐
150€ - 200€ ☐
200+€ ☐
